# Synthesis, Spectral Characterization and Crystals Structure of some Arsane Derivatives of Gold (I) Complexes: A Comparative Density Functional Theory Study

**DOI:** 10.1371/journal.pone.0119620

**Published:** 2015-03-23

**Authors:** Omar bin Shawkataly, Chin-Ping Goh, Abu Tariq, Imthyaz Ahmad Khan, Hoong-Kun Fun, Mohd Mustaqim Rosli

**Affiliations:** 1 Chemical Sciences Programme, School of Distance Education, Universiti Sains Malaysia, 11800, Penang, Malaysia; 2 X-ray Crystallography Unit, School of Physics, Universiti Sains Malaysia, 11800, Penang, Malaysia; 3 Department of Pharmaceutical Chemistry, College of Pharmacy, King Saud University, Riyadh, 11451, Kingdom of Saudi Arabia; University of Calgary, CANADA

## Abstract

A series of complexes of the type LAuCl where L = tris(*p*-tolylarsane), tris(*m*-tolylarsane), bis(diphenylarsano)ethane, and tris(naphthyl)arsane have been synthesized. All of the new complexes, **1-4**, have been fully characterized by means of ^1^H NMR and ^13^C NMR spectroscopy and single crystal X-ray crystallography. The structures of complexes **1-4** have been determined from X-ray diffraction data. The linear molecules have an average bond distance between gold-arsenic and gold-chlorine of 2.3390Å and 2.2846Å, respectively. Aurophilic interaction was prominent in complex **1** and **3**, whereas complex **2** and **4** do not show any such interaction. The intermolecular gold interaction bond length was affected by the electronegativity of the molecule. The computed values calculated at DFT level using B3LYP function are in good agreement with the experimental results.

## Introduction

Gold (I) compounds have a great potential role in biological and medical chemistry. [1,2]. Over the past ten years, the application of gold complexes in the development of antitumor or anticancer drugs have gained the interest of many researchers [3–7]. While numerous crystallographic studies of organogold(I) complexes of phosphines have been reported in the last few years, chemical reactions of arsenic derivatives with gold remained largely unexplored, and there are only a few reactions reported earlier [8–16]. The construction of unusual molecular geometry is aided by the tendency of gold(I) to form linear, 2-coordinate complexes that may then undergo additional weaker aurophilic attractions. If the gold(I) centres are bonded to bidentate ligands, polymers or rings may be formed, and the Au—-Au interactions can determine the favored structure [17–19].

Part of our interest in gold(I) complexes with group 15 ligands [20–21] has led us to synthesize, characterize and determine the molecular structure of some gold(I) complexes with tertiary arsine ligands. The feasibility of calculating the theoretical optimized structures of the synthesized compounds are part of the research interest as well.

## Materials and Methods

### 2.1 Chemicals, starting materials and spectroscopic measurements

All starting materials were used as received from commercial sources, and the solvents such as methanol, ethanol and hexane were obtained from QRec Chemicals Ltd., and were purified according to conventional methods. H[AuCl_4_] (49%) and dimethyl sulfide was purchased from Sigma-Aldrich Chemical Co. (Germany) and used as received. The melting points of the compounds were recorded in open capillaries using a UK manufactured Bibby Scientific Ltd. Stuart Melting Point Apparatus, and were uncorrected. Deuterated chloroform (CDCl_3_) was purchased from Aldrich and used as a solvent for ^1^H NMR and ^13^C NMR. These NMR studies were carried out on a Bruker Shield B2H 400 FT-NMR spectrometer using 5 mm tubes. The ^1^H NMR and ^13^C NMR chemical shifts were referenced against tetramethylsilane (TMS). A U.S. manufactured Perkin Elmer Series II CHNS/O Analyzer 2400, was used for the microanalysis of complexes.

### 2.2 General procedure for the synthesis of complexes 1–4

The complexes **1–4** were prepared as colorless, air-stable products by treating one mole equivalent of AuCl(SMe_2_) obtained as per the conventional method [22], with the corresponding arsenic ligand in dichloromethane at room temperature. The solution was stirred at room temperature for about 2 h. The reaction was checked for completion by thin layer chromatography (TLC) and the solvent was removed under pressure to give an oily mass; a white solid is obtained from the treatment of the oily mass with diethylether.

### 2.3. X-ray structural determination

Determination of cell constants and data collection were carried out at 100.0(1) K using the Oxford Cryosystem Cobra low-temperature attachment with Mo Kα radiation at (λ = 0.71073) on a Bruker SMART APEX2 CCD area-detector diffractometer equipped with a graphite monochromator [23]. The data was reduced using SAINT [23]. A semi-empirical absorption correction was applied to the data using SADABS [23]. The structures were solved by direct methods and refined against F^2^ by full-matrix least-squares using SHELXTL [24]. Hydrogen atoms were placed in calculated positions.

### 2.4. Computational details

The optimized geometric structures of four gold (I) complexes had been calculated using the DFT method. The gradient corrected Becke’s three parameter hybrid exchange function in combination with the correlation function of Lee, Yang and Parr (B3LYP) [25–27] as implemented in the software package Gaussian 03 was used [28]. Geometry optimization procedures were started from the experimental crystallographic data of the complexes employing the 6–311G basis set for H, C, Cl and As atoms. For Au atoms, the LANL2 double zeta basis set with effective core potentials (ECP) that represent 60 core electrons and 19 valence electrons were used [29]. The larger size basis set such as the Stuttgart relativistic small-core (RSC) 1997 ECP and LANL2 triple zeta ECP are also tested for Au atoms [30–31].

## Results and Discussion

### 3.1 Synthesis and characterization

A series of arsane-substituted gold(I) complexes, (*p*-tolyl)_3_AsAuCl **1**, (*m*-toly)l_3_AsAuCl **2**, *bis*(diphenylarsanyl)Au_2_Cl_2_ **3**, and (naphthyl)_3_AsAuCl **4** were synthesized. Complexes **1**–**4** were prepared for comparison with the previously reported adduct Ph_3_AsAuCl [15] and (*o*-tolyl)_3_AsAuCl [32]. All of the new compounds have been characterized by elemental analysis; ^1^H NMR and ^13^C NMR where appropriate. The physical properties, ^1^H NMR and ^13^C NMR data of complexes **1**–**4** are tabulated in Table 1 and Table 2.

**Table 1:** Physical state, melting point, elemental analysis (EA) and yield data of compounds **1–4**.

Compd.	Physical state	m.p./ °C (dec.)	EA (%, Calcd.)	Yield (%)
**1**	Colorless, needle-like cryst.	189	C 42.92 (43.43), H 1.77 (3.64)	88
**2**	Colorless, block shaped cryst.	196	C 43.66 (43.43), H 2.26 (3.64)	90
**3**	Colorless, needle-like cryst.	235	C 33.08 (32.83), H 2.03 (2.54)	90
**4**	Colorless, needle-like cryst.	252	C 48.62 (52.31), H 1.53 (3.07)	95

**Table 2:** Experimental ^1^H and ^13^C NMR data of compounds **1–4**.

Compound	^1^H NMR data	^13^C NMR data
**1**	7.4–7.2 (m, 12H, phenyl); 2.4 (s, 9H, CH_3_)	140–129 (Ph); 21.8 (CH_3_)
**2**	7.4–7.2 (m, 12H, phenyl); 2.4 (s, 9H, CH_3_)	142–128 (Ph); 21.8 (CH_3_)
**3**	7.6–7.3 (m, 20H, phenyl); 2.6 (s, 4H, CH_2_)	133–129 (Ph); 24.3 (CH_2_)
**4**	8.0–7.3 (m, 21H,naphthyl)	135–125 (Ph)

The microanalyses of all the synthesized complexes agreed with the proposed molecular formulae within the experimental errors. The ^1^H NMR spectra of **1, 2** and **3** showed a multiplet of around δ 7.2–7.6 ppm, characteristic of phenyl groups. For the methyl groups, a singlet was observed at δ 2.4 for the tolyl substituted complexes. In the case of **4**, aromatic protons were downfield and observed at higher δ values, at 7.3–8.0 ppm. ^13^C NMR spectra of all the substituted clusters showed prominent signals at around δ 125–142 ppm, characteristic of phenyl carbons. In addition, the methyl carbons appeared at δ 21 ppm for the complexes **1** and **2**, and at δ 24 ppm for complex **3**.

#### 3.2.1 X-Ray crystal structure analysis

**Fig 1:**
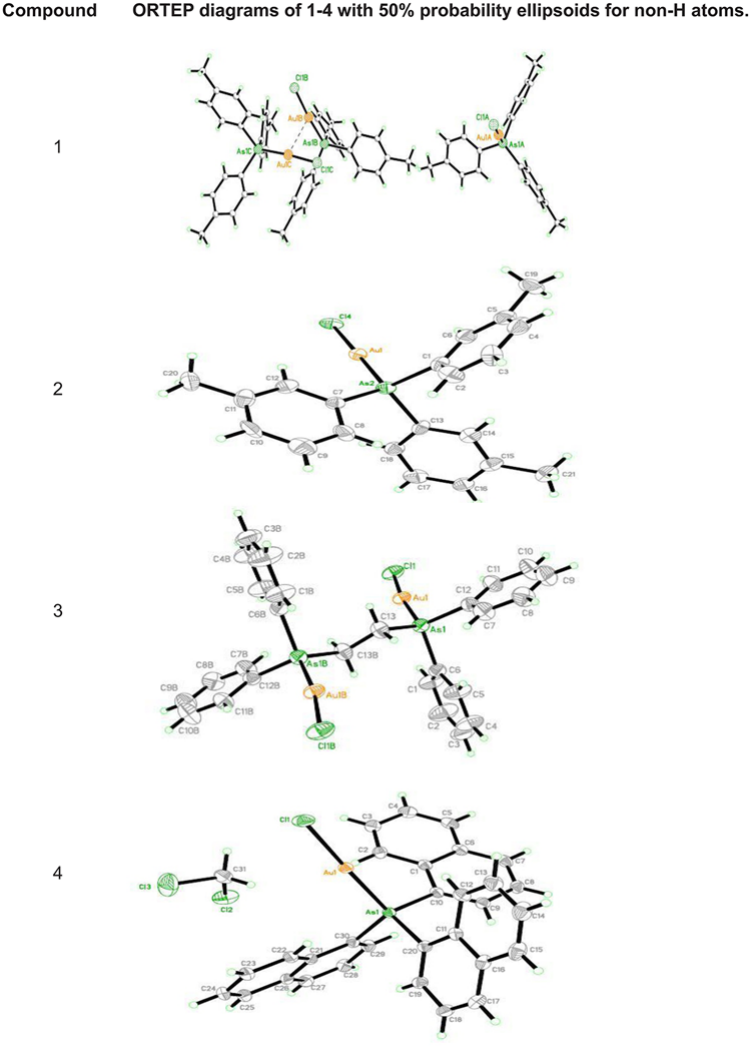
The ORTEP diagrams of 1–4 with 50% probability ellipsoids for non-H atoms.

Crystals of **1**–**4** were obtained by slow diffusion dichloromethane solutions with methanol; see Table 3 for crystal data. ORTEP representations of the molecular structures are shown in Fig. 1. Table 4 summarizes important bond lengths found for these complexes. All the complexes contain almost linear geometry between As-Au-Cl atoms.

**Table 3:** Crystallographic parameters for compounds **1–4**.

Compound	1	2	3	4
Empirical formula	C21H21AsAuCl	C_21_H_21_AsAuCl	C_26_H_24_As_2_Au_2_Cl_2_	C_30_H_21_AsAuCl.CH_2_Cl_2_
Fw	580.72	580.72	951.15	773.73
Colour, habit	Colourless, block	Colourless, needle	Colourless, needle	Colourless, block
Crystal system	Monclinic	Orthorhombic	Monoclinic	Monclinic
Space group	P2_1_	Pnma	C2/c	P2_1_/c
Crystal size/mm3	0.23 x 0.23 x 0.43	0.51 x 0.13 x 0.08	0.08 x 0.09 x 0.59	0.10 x 0.17 x 0.49
a/ Å	12.9884(9)	11.6328(11)	17.4293(4)	9.4715(3)
b/ Å	17.0042(12)	11.9721(11)	14.8826(4)	18.0504(5)
c/ Å	13.3251(9)	14.0632(12)	11.5267(3)	16.0002(5)
α/°	90.00	90.00	90.00	90.00
β/°	90.318(2)	90.00	116.607(2)	90.461(1)
γ/°	90.00	90.00	90.00	90.00
Volume/ Å^3^	2942.9(4)	1958.6(3)	2673.31(13)	2718.09(14)
T/K	100(1)	100(1)	100(1)	100(1)
Z	6	4	4	4
Dcalcd/mg m^-3^	1.966	1.969	2.363	1.891
λ/ Å	0.71073	0.71073	0.71073	0.71073
μ(Mo-Ka)/mm^-1^	9.308	9.324	13.663	6.935
F(000)	1656	1104	1752	1488
θ range/°	2.2–27.5	2.4–30.0	1.9–30.2	1.7–37.4
Reflections tot, unique, Rint	41335, 13189, 0.048	12848, 5537, 0.041	27755, 3949, 0.041	52853, 14091, 0.040
Nref, Npar	13189, 646	5537, 171	3949, 145	14091, 325
Flack x	0.019(8)	0.5(2)		
R1,wR2	0.0458/0.1199	0.0429/0.1108	0.0280/0.0728	0.0287, 0.0665
Largest diff. peak, hole/e Å^-3^	6.79, -1.23	1.94, -2.30	1.56, -0. 64	3.05, -1.14
GOF	1.02	1.15	1.03	1.021

**Table 4:** Bond distances (Å) in some Au(L)Cl complexes.

M	L	Au-As	Au-Cl	Reference
Au	AsPh_3_	2.334(3)	2.280(8)	[15]
Au	As(*o*-tolyl)_3_	2.3443(15)	2.3475(15)	[32]
Au	As(*p*-tolyl)_3_	2.3327(4);	2.2794(8);	a
		2.3450(9);	2.2969(3);	
		2.3414(1)	2.2814(5)	
Au	As(*m*-tolyl)_3_	2.3292(2)	2.2852(7)	a
Au	Bis(diphenylarsino) ethane	2.3422(3);	2.2893(3);	a
		2.3423(0)	2.2892(9)	
Au	As(naphthyl)_3_	2.3402(5)	2.2706(7)	a

^a^Present work

The gold complex, [(*p*-tolyl_3_As)AuCl] **1** contains three independent molecules in the asymmetric unit. All three molecules exhibit the expected linear geometry at the gold atom. The Au—As and Au—Cl bond possess lengths of 2.3327(4)/2.2794(8)Å, 2.3450(9)/2.2969(3)Å and 2.3414(1)/2.2814(5)Å, respectively. They are comparable to those reported for [(*o*-tolyl_3_As)AuCl] and [(Ph_3_As)AuCl] [32, 15]. The complex, [(*m*-tolyl_3_As)AuCl] **2** is an independent molecule with a linear geometry at the gold atom. The obtained Au—As and Au—Cl bond length of 2.3292(2)/2.2852(7)Å is comparable to those reported for [(*o*-tolyl_3_As)AuCl] and [(Ph_3_As)AuCl] [32, 15].

The asymmetric unit of gold complex, [C_26_H_24_As_2_Au_2_Cl_2_] **3** consists of half a molecule and the other half is symmetry generated. The molecule in complex **3** exhibits the expected linear geometry at the gold atom. The Au—As and Au—Cl bond lengths of 2.3422(3)/2.2893(3)Å and 2.3423(0)/2.2892(9)Å found in complex **3** are in agreement to the bond lengths reported in [(Ph_3_As)AuCl] [15] and [(*o*-tolyl)_3_AsAuCl] [32]. The complex, [(naphthyl_3_As)AuCl.CH_2_Cl_2_] **4** is an independent molecule with a linear geometry at the gold atom. The Au—As and Au—Cl bond lengths are found to be 2.3402(5)/2.2706(7)Å are comparable to those reported for [(Ph_3_As)AuCl] [32] and [(o-tolyl)_3_AsAuCl] [15] as well.

#### 3.2.2 Aurophilic interactions

The identification of Au—-Au interactions for compounds **1**–**4**, using the Contacts functionality of Mercury CSD 3.3.1 software found that only molecules of complex **1** and **3** showed Au—-Au interactions of 3.266Å and 3.158Å, respectively. This is comparable to the result reported by Bott RC et al. where an aurophilic interaction of (3.375(1)Å) was observed in the [(*p*-tolyl)_3_PAuCl] complex [33] and also those reported by Lim SH et al., where the Au—-Au interactions within the range of 3.1163(2)-3.1668(3)Å were observed for the complex of Au_2_(*μ*-dpae)Cl_2_ [dpae is 1,2-bis(diphenylarsino)ethane] [34]. Short Au—-Au contacts, both inter- and intra-molecular, are quite common in gold(I) chemistry, for example in the bridged phosphine complex cis[(AuCl),dppen] [dppen is 1,2-bis(diphenylphosphino)ethene] [35] there are intra-molecular Au—-Au contacts of 3.05(1)Å. Intermolecular contacts in [(AuCl)_2_dppp], [dppp is 1,3-bis(diphenylphosphino) propane] is 3.316Å between adjacent molecules related by the b glide [36] meaning that the structure may be considered as being composed of polymeric chains. The propensity of gold(I) atoms to aggregate via inter- and intra-molecular aurophilic interactions often provides additional stabilization to such complexes. This often affords interesting and unpredictable extended coordination structures in the molecular structure of these types of complexes [37–39].

### 3.3 Geometrical parameters

**Fig 2:**
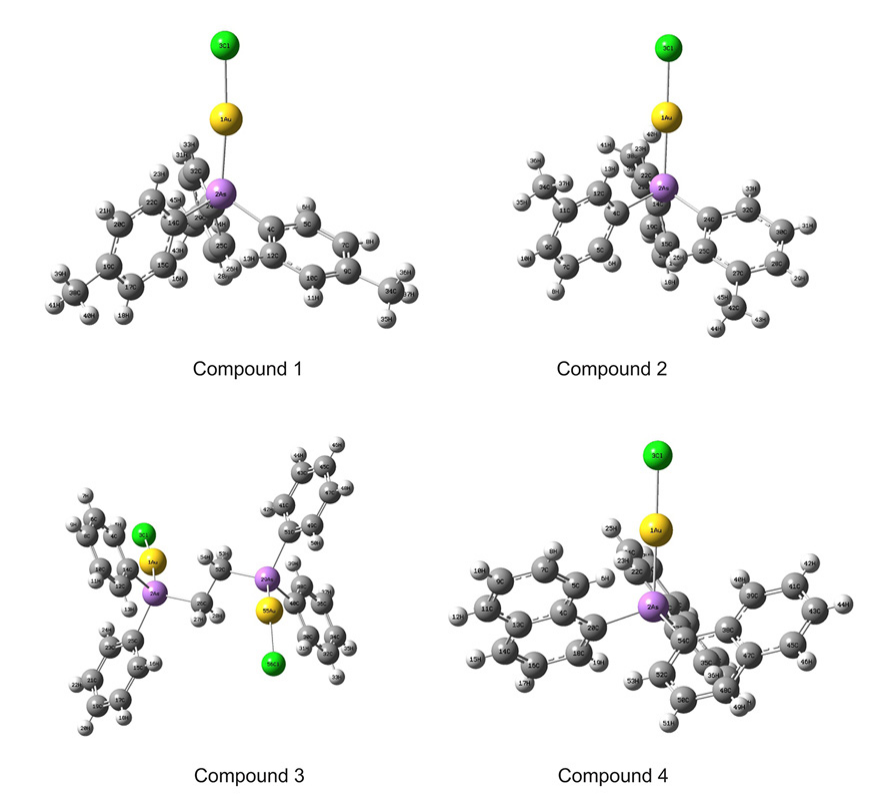
Numbering system adopted in the geometrical structures of compounds 1–4.

The atom numbering scheme for compounds **1**–**4** are given in Fig. 2. The optimized bond lengths, bond angles and dihedral angles with LANL2DZ ECP basis set for Au atoms and 6–311(G) basis set for H, C, Cl and As atoms are tabulated in Table 5. The biggest difference between the computed and the experimental bond lengths are at Au1-Cl3 for each compound, with an average of 0.123 ±0.011 Ǻ. This deviation between the calculated and the experimental bond lengths are probably due to the reason of relativistic bond length contractions [40]. The importance of relativistic effects in gold chemistry had been reported by Pitzer [41], *Pyykkö* and Desclaux in late 1970s [42]. Schwerdtfeger et al. had reported specifically the relativistic effects in Gold (I) complexes using multi-electron adjusted non-relativistic and relativistic pseudopotentials Hartree-Fock (HF) method [43]. According to *Pyykkö* [40], the studies conducted by Ziegler et al. [44] and Hay et al. [45] revealed that the relativistic bond lengths contraction for Au-Cl were 0.164 Ǻ and 0.13 Ǻ, respectively.

**Table 5:** Selected bond lengths (Å), bond angles (°) and dihedral angles (°) of experimental (Exp.) and calculated (Calcd.) geometrical parameters of compounds **1–4**.

Bond length (Å)	Exp.	Calc.	Bond length (Å)	Exp.	Calc.	Bond length (Å)	Exp.	Calc.	Bond length (Å)	Exp.	Calc.
Au1-As2	2.344	2.429	Au1-As2	2.329	2.429	Au1-As2	2.342	2.429	Au1-As2	2.340	2.439
Au1-Cl3	2.295	2.411	Au1-Cl3	2.285	2.406	Au1-Cl3	2.289	2.405	Au1-Cl3	2.271	2.410
As2-C4	1.939	1.951	As2-C4	1.993	1.955	Au55-As29	2.342	2.429	As2-C20	1.934	1.968
As2-C14	1.942	1.952	As2-C14	1.871	1.955	As2-C14	1.930	1.954	As2-C37	1.938	1.968
As2-C24	1.932	1.953	As2-C24	1.938	1.955	As2-C25	1.936	1.951	As2-C54	1.938	1.968
C4-C5	1.401	1.401	C4-C5	1.391	1.401	As2-C26	1.956	1.984	C20-C18	1.377	1.382
C14-C15	1.389	1.401	C14-C15	1.390	1.401	As29-C40	1.930	1.954	C37-C35	1.370	1.382
C24-C25	1.419	1.401	C24-C25	1.390	1.398	As29-C51	1.936	1.951	C54-C52	1.377	1.382
						As29-C52	1.956	1.983			

Bond angle (°)	Exp.	Calc.	Bond angle (°)	Exp.	Calc.	Bond angle (°)	Exp.	Calc.	Bond angle (°)	Exp.	Calc.
Cl3-Au1-As2	175.1	179.7	Cl3-Au1-As2	178.7	178.9	Cl3-Au1-As2	172.5	176.7	Cl3-Au1-As2	177.9	179.5
As2-C4-C5	116.7	118.8	As2-C4-C5	120.0	121.9	As2-C14-C4	119.1	119.0	As2-C20-C18	118.9	118.6
As2-C14-C15	118.7	122.1	As2-C14-C15	120.2	121.7	As2-C25-C15	121.9	121.9	As2-C37-C35	118.4	118.6
As2-C24-C25	123.4	122.1	As2-C24-C25	123.2	121.7	Au1-As2-C26	119.0	111.1	As2-C54-C52	118.8	118.6
C7-C9-C34	118.8	120.9	C12-C11-C34	119.3	120.3	Cl56-Au55-As29	172.5	176.8	C11-C13-C14	120.7	121.2
C17-C19-C38	118.4	120.7	C22-C21-C38	120.8	120.3	As29-C40-C30	119.1	121.2	C28-C30-C31	120.9	121.2
C27-C29-C42	121.9	120.7	C25-C27-C42	121.3	120.3	As29-C51-C41	121.9	121.9	C45-C47-C48	121.4	121.2

Dihedral angle (°)	Exp.	Calc.	Dihedral angle (°)	Exp.	Calc.	Dihedral angle (°)	Exp.	Calc.	Dihedral angle (°)	Exp.	Calc.
Au1-As2-C4-C5	-70.8	-35.8	Au1-As2-C4-C5	155.8	144.6	Au1-As2-C14-C12	169.1	170.4	Au1-As2-C20-C18	124.5	130.7
Au1-As2-C14-C15	168.9	145.1	Au1-As2-C14-C15	162.8	147.4	Au55-As29-C40-C38	-169.1	-170.6	Au1-As2-C37-C35	132.3	131.1
Au1-As2-C24-C25	157.9	144.2	Au1-As2-C24-C25	122.0	143.1	As29-C52-C26-As2	180.0	180.0	Au1-As2-C54-C52	130.6	131.1

Calculations for the gold complexes using Stuttgart RSC 1997 ECP and LANL2TZ ECP for Au atoms were conducted for this work with the objective to investigate if there are improvements for the computed bond length values at Au1-Cl3. The optimized computed bond lengths are tabulated in Table 6. It was found that the difference between the computed and the experimental bond lengths at Au1-Cl3 were reduced at an average value of 0.018 Ǻ and 0.031 Ǻ, respectively when using the basis set of Stuttgart RSC 1997 ECP and LANL2TZ ECP for Au atoms. Odoh et al reported the vibrational frequencies and reaction enthalphy changes of several uranium (VI) compounds computed using the Stuttgart small-core and large-core relativistic effective core potentials (RECP) compared to those obtained using DFT and a four-component one-electron scalar relativistic approximation of the full Dirac equation with large all electron (AE) bases set [46]. From the study it was claimed that the small-core RECP show better agreement with the four-component scalar-relativistic AE method than the large-core RECP. However, the results in Table 6 shown that the improvement obtained by using Stuttgart RSC ECP 1997 basis set was not as good as that obtained by using the non-relativistic LANL2TZ basis set. The calculation for compound **4** also terminated pre-maturely due to convergence failure.

**Table 6:** Selected bond lengths (Å) of experimental (Exp.) and calculated (I, LANL2DZ), (II, Stuttgart RSC 1997 ECP) and (III, LANL2TZ) bond lengths (Å) of compounds **1–4**.

Compound 1	Exp.	I	II	III	Δ(I-Exp.)	Δ(II-Exp.)	Δ(III-Exp.)
Au1-As2	2.344	2.429	2.433	2.410	0.085	0.089	0.066
Au1-Cl3	2.295	2.411	2.390	2.379	0.115	0.095	0.084
As2-C4	1.939	1.951	1.954	1.952	0.013	0.016	0.014
As2-C14	1.942	1.952	1.955	1.953	0.010	0.013	0.011
As2-C24	1.932	1.953	1.955	1.954	0.020	0.023	0.022
C4-C5	1.401	1.401	1.403	1.402	0.000	0.002	0.001
C14-C15	1.389	1.401	1.403	1.403	0.013	0.014	0.014
C24-C25	1.419	1.401	1.400	1.400	-0.019	-0.019	-0.019

Compound 2	Exp.	I	II	III	Δ(I-Exp.)	Δ(II-Exp.)	Δ(III-Exp.)
Au1-As2	2.329	2.429	2.431	**2.411**	0.100	0.102	0.082
Au1-Cl3	2.285	2.406	2.389	2.375	0.120	0.104	0.090
As2-C4	1.993	1.955	1.957	1.956	-0.038	-0.036	-0.037
As2-C14	1.871	1.955	1.957	1.955	0.083	0.086	0.084
As2-C24	1.938	1.955	1.958	1.956	0.017	0.020	0.018
C4-C5	1.391	1.401	1.401	1.401	0.010	0.010	0.010
C14-C15	1.390	1.401	1.401	1.401	0.011	0.011	0.011
C24-C25	1.390	1.398	1.398	1.398	0.008	0.008	0.008

Compound 3	Exp.	I	II	III	Δ(I-Exp.)	Δ(II-Exp.)	Δ(III-Exp.)
Au1-As2	2.342	2.429	2.432	2.410	0.087	0.090	0.068
Au1-Cl3	2.289	2.405	2.387	2.374	0.116	0.098	0.085
Au55-As29	2.342	2.429	2.432	2.410	0.086	0.090	0.068
As2-C14	1.930	1.954	1.957	1.955	0.024	0.027	0.025
As2-C25	1.936	1.951	1.954	1.952	0.015	0.018	0.016
As2-C26	1.956	1.984	1.984	1.984	0.028	0.028	0.028
As29-C40	1.930	1.954	1.957	1.955	0.024	0.027	0.025
As29-C51	1.936	1.951	1.953	1.952	0.015	0.017	0.016
As29-C52	1.956	1.983	1.984	1.984	0.028	0.028	0.028

Compound 4	Exp.	I	II*	III	Δ(I-Exp.)	Δ(II-Exp.)	Δ(III-Exp.)
Au1-As2	2.340	2.439	2.447	2.423	0.099	0.107	0.083
Au1-Cl3	2.271	2.410	2.394	2.379	0.139	0.123	0.108
As2-C20	1.934	1.968	1.970	1.969	0.034	0.036	0.035
As2-C37	1.938	1.968	1.970	1.969	0.030	0.032	0.031
As2-C54	1.938	1.968	1.970	1.969	0.030	0.032	0.031
C20-C18	1.377	1.382	1.383	1.382	0.006	0.006	0.005
C37-C35	1.370	1.382	1.382	1.382	0.013	0.012	0.012
C54-C52	1.377	1.382	1.383	1.382	0.005	0.006	0.005

*Premature termination of calculation.

Pantazis et al. studied the use of a family of segmented all-electron relativistically contracted (SARC) basis set for the third-row transition metal atoms. It was reported that the level of agreement between the experimental and computed value was successful. For further investigation on the relativistic effects of gold (I) complexes, this could be an alternative approach to be considered [47].

**Fig 3:**
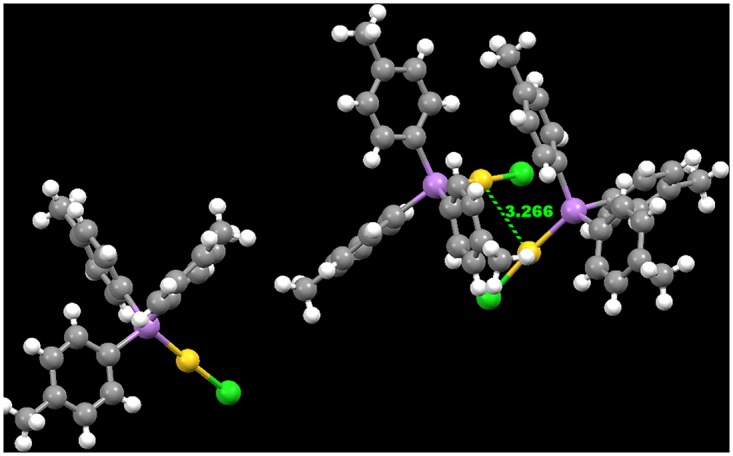
The Au-Au interaction in an asymmetric unit of compound 1.

According to Crespo [48], the typical linear coordination at the gold centres is frequently distorted by the presence of the aurophilic interactions. Comparing the bond angles at Cl-Au-As for compounds **1**–**4**, it was found that complexes **1** and **3** with Au—-Au interaction were found distorted slightly more compared to those without Au—-Au interactions. The differences between the experimental and the calculated bond angles for compounds **1** and **3** are 4.6° and 4.21°, respectively. However, there were only at 0.17° and 1.64° for compounds **2** and **4**, respectively. This is because the effects of the intermolecular Au—-Au interactions that occurred in complexes **1** and **3** were not taken into consideration during the computational molecular structure optimization job. Besides this, such aurophilic interactions in complexes **3** had also caused the biggest difference between the experimental and calculated values of bond angle at the position of Au1-As2-C26. The calculated bond angle is 7.96° smaller than the X-ray values found. Looking at the dihedral angle value for complex 1 at Au1-As2-C4-C5, it was also found that there was a large difference between the computed value and the experimental value. Looking down from the direction of C4-As2 bond, the dihedral angle is 35° distorted compared to the computed value. As it was shown in Fig. 3, due to Complex **1** contains three independent molecules in the asymmetric unit. The relatively strong Au-Au interaction had cause two inter-molecules to get closer. In order to compromise for the steric strain, the X-ray structure was deviate from the ideal gas phase solid state computed structure. According to Jensen [49], energy needed for distorting a molecule by rotation around a bond is often lower compared to stretching or bending a bond.

#### 3.3.1 Ligand effects on the Au---Au interactions

**Fig 4:**
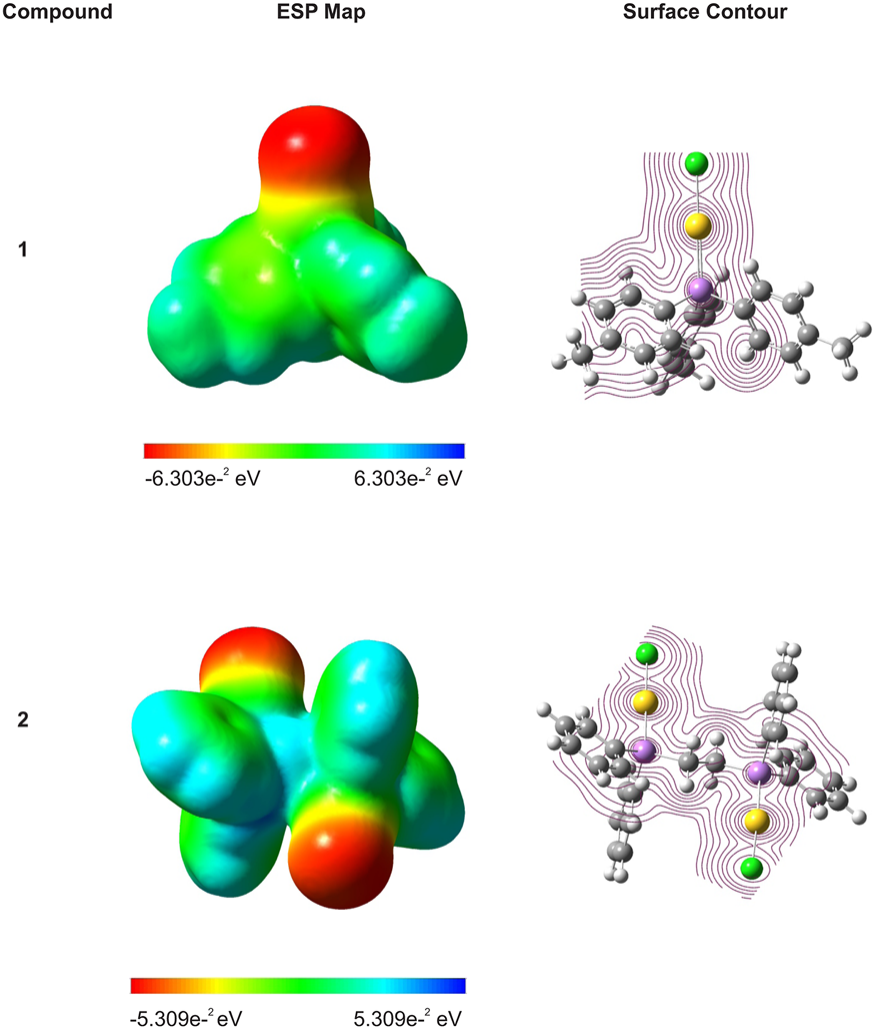
The electrostatic potential (ESP) map and surface contours of compounds 1 and 3.

Gold centres tend to get closer to one another. The principle is the less the steric hindrance of the ligand, the higher the dimensionality of the aggregation that normally works, as a general rule in the formation of the final species [48]. Aurophilic interactions, with Au—-Au distances ranging from 2.9 to 3.32 Å are thought to arise from a combination of relativistic and correlation effects and have been shown to have interaction energy of between 20 and 50kJ/mol, which is comparable with that of a hydrogen bond [50]. Schwerdfeger et al. reported that relativistic bond stabilizations or destabilizations are dependent on the electronegativity of the ligand, showing the largest bond destabilization for AuF (86kJ/mol) and the largest stabilization for AuLi (-174kJ/mol) [51]. As discussed earlier, we observed that the Au—-Au bond lengths for compounds **1** and **3** are of 3.266Å and 3.158Å, respectively. Carrying out the calculation for the electrostatic potential (ESP) map for the ligands of compounds **1** and **3**, it was found that the electronegativity values are-2.702e^-2^ eV and-2.520e^-2^ eV for the ligands in **1** and **3** respectively. Using Stuttgart relativistic small-core ECP basis set for Au atoms to determine the ESP for compound **1** and **3**, it was also found that the overall electronegativity determined from the ESP map are-6.303e^-2^ eV and-5.309e^-2^ eV respectively. Thus, we believe the longer bond distance in the intermolecular Au—-Au interaction is due to the stronger electronegativity of the ligand as well as the overall electronegativity of the individual molecule. Fig. 4 shows the ESP map and the surface contours for compounds **1** and **3**, where the potential increase in the order of red<orange<yellow<green<blue.

#### 3.3.2 Predicting NMR properties

The optimized geometries of compounds **1**–**4** that were obtained earlier using LANL2DZ/6–3111G functions had been utilized for the prediction of the nuclear magnetic shielding tensors using the gauge-including atomic orbital (GIAO) methods. The predicted shielding tensor values are listed in Table 7. For comparison purpose, IGLO II basis set was utilized for H and C atom in the NMR chemical shift calculation of Compound **3**. It was observed that, the computed values of ^1^H NMR were closer to the experimental value when the IGLO II basis set was used for H and C atoms during the optimization calculation [30–31]. However, the computed values of ^13^C NMR were closer to the experimental value when the 6–311G basis set was utilized for H and C atoms during the optimization calculation.

**Table 7:** Calculated ^1^H and ^13^C NMR data of compounds **1–4**.

Compound	Calculated ^1^H NMR data	Calculated ^13^C NMR data
**1**	6.7–5.8 (12H); 1.7–1.0 (9H)	142–127 (18C); 15.9–15.6 (3C)
**2**	6.8–5.7 (12H); 1.8–0.9 (9H)	141–127 (18C); 15.8–15.5 (3C)
**3**	a6.7–6.4 (20H); 1.5 (2H); 0.4 (2H)	136–128 (24C); 24.6 (2C)
	b7.8–7.3 (20H); 2.5 (2H); 1.2 (2H)	143–134 (24C); 31.2 (2C)
**4**	7.3–5.8 (21H)	135–123 (30C)

^a^ Using 6–311G basis set for H and C atoms

^b^ Using IGLO II basis set for H and C atoms

**Fig 5:**
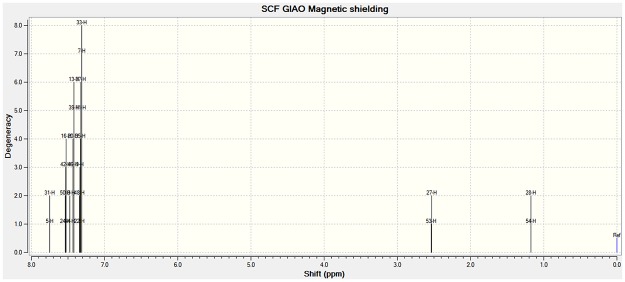
Calculated ^1^H nuclear magnetic tensors shielding value of compound 3.

The experimental ^1^H NMR chemical shift for compound **3** shows that there is a singlet at δ 2.6 ppm position, this indicates that all proton in each methylene group are equivalent. However, the calculated ^1^H nuclear magnetic tensor shielding value shows that there are two separated chemical shifts at 2.5 ppm and 1.2 ppm. The peak at 2.5 ppm area refers to the atoms of H27 and H53 (see Fig. 5). The deviation between the experimental and computed results is mainly due to the computed values being absolute chemical shielding tensors that do not take into consideration of the effects of the sample solvent and temperature. Cabrera et al. reported that the ^1^H-NMR spectrum of Ru_4_(CO)_9_(μ-CO){μ_4_-η^2^-PCH_2_CH_2_P(C_6_H_5_)_2_}(μ_4_-η^4^-C_6_H_4_) showed a non-rigidity of the CH_2_-CH_2_ bridge. Originally the protons in each methylene group are equivalent at room temperature. However, on cooling the CH_2_ signals are broadened to finally give four lines at 3.49, 3.03, 2.98 and 2.23 ppm [52]. The experimental NMR results obtained here were from room temperature analysis, and it was expected that the same sample analysis conducted at a lower temperature would separate the single peak into two or more peaks.

## Conclusion

Several gold (I) complexes were synthesized with different arsane ligands. All the four reported complexes show linear geometry of the As-Au-Cl bond. The bond lengths between As-Au and Au-Cl were found to be similar to earlier reported structures. Au—-Au interaction was prominent in complex **1** and **3**, whereas complex **2** and **4** do not show any such interaction. The computed geometric values obtained at DFT level using B3LYP function with the basis set combination of LANL2DZ ECP/6–311G are in good agreement with the experimental results. ^1^H and ^13^C NMR chemical shift were also calculated using GIAO method. However, ^1^H NMR computed values are in good agreement with the experimental values when IGLO II basis set was used for H and C atoms. Whereas, ^13^C NMR computed values are in good agreement with the experimental values when 6–311G basis set was used for H and C atoms during geometry optimization.

## Supplementary Data

CCDC numbers 823552, 823553, 823554 and 823555 contain the supplementary crystallographic data for **1–4** (see S1–S4 Data and S1–S4 Text). These data can be obtained free of charge via http://www.ccdc.cam.ac.uk/conts/retrieving.html, or from the Cambridge Crystallographic Data Centre, 12 Union Road, Cambridge CB2 1EZ, UK; fax: (+44) 1223–336–033; or e-mail: deposit@ccdc.cam.ac.uk.

## Supporting Information

S1 Data Compound 1 crystallographic information file. (CIF)

S2 Data Compound 2 crystallographic information file. (CIF)

S3 Data Compound 3 crystallographic information file. (CIF)

S4 Data Compound 4 crystallographic information file. (CIF)

S1 Text Compound 1 checkcif file. (PDF)

S2 Text Compound 2 checkcif file. (PDF)

S3 Text Compound 3 checkcif file. (PDF)

S4 Text Compound 4 checkcif file. (PDF)
