# Supplementary material for: Synthesis, Spectral Characterization and Crystals Structure of some Arsane Derivatives of Gold (I) Complexes: A Comparative Density Functional Theory Study
Source: PLoS One. 2015 Mar 23;10(3):e0119620. doi: 10.1371/journal.pone.0119620 (PMC4370652; doi:10.1371/journal.pone.0119620)

checkCIF/PLATON report

Structure factors have been supplied for datablock(s) I

THIS REPORT IS FOR GUIDANCE ONLY. IF USED AS PART OF A REVIEW PROCEDURE FOR PUBLICATION, IT SHOULD NOT REPLACE THE EXPERTISE OF AN EXPERIENCED CRYSTALLOGRAPHIC REFEREE.

No syntax errors found.   CIF dictionary   Interpreting this report

Datablock: I

```
Bond precision:  As- C = 0.0080 A        Wavelength=0.71073

Cell:           a=11.6328(11)     b=14.0632(12)     c=11.9721(11)
                alpha=90          beta=90           gamma=90
Temperature:    100 K

                Calculated                Reported
Volume          1958.6(3)                 1958.6(3)
Space group     P n m a                   P n m a
Hall group      -P 2ac 2n                 -P 2ac 2n
Moiety formula  C21 H21 As Au Cl          ?
Sum formula     C21 H21 As Au Cl          C21 H21 As Au Cl
Mr              580.72                    580.71
Dx,g cm-3       1.969                     1.969
Z               4                         4
Mu (mm-1)       9.324                     9.324
F000            1104.0                    1104.0
F000'           1097.59
h,k,lmax        16,19,16                  16,19,16
Nref            2967                      2949
Tmin,Tmax       0.232,0.479               0.087,0.526
Tmin'           0.008

Correction method= MULTI-SCAN

Data completeness= 0.994          Theta(max)= 30.000

R(reflections)= 0.0375( 2413)     wR2(reflections)= 0.0911( 2949)

S = 1.148                   Npar= 194
```

The following ALERTS were generated. Each ALERT has the format
test-name_ALERT_alert-type_alert-level.
Click on the hyperlinks for more details of the test.

Alert level C

```
PLAT048_ALERT_1_C MoietyFormula Not Given ........................     Please Do !
PLAT911_ALERT_3_C Missing # FCF Refl Between THmin & STh/L=  0.600         17 Report
PLAT913_ALERT_3_C Missing # of Very Strong Reflections in FCF ....          3 Note
PLAT971_ALERT_2_C Check Calcd Residual Density  0.80A From     Au1       2.03 eA-3
PLAT971_ALERT_2_C Check Calcd Residual Density  0.82A From     Au1       1.85 eA-3
PLAT972_ALERT_2_C Check Calcd Residual Density  0.84A From     Au1      -2.49 eA-3
```

Alert level G

```
PLAT083_ALERT_2_G SHELXL Second Parameter in WGHT Unusually Large.      14.42 Report
PLAT093_ALERT_1_G No su’s on H-positions, refinement reported as .      mixed
PLAT171_ALERT_4_G The CIF-Embedded .res File Contains EADP Records          2 Report
PLAT301_ALERT_3_G Main Residue  Disorder ............ Percentage =         79 Note
PLAT380_ALERT_4_G Incorrectly? Oriented X(sp2)-Methyl Moiety .....        C14 Check
PLAT432_ALERT_2_G Short Inter X...Y Contact  C2     ..  C13X    ..       2.50 Ang.
PLAT432_ALERT_2_G Short Inter X...Y Contact  C2     ..  C12X    ..       2.85 Ang.
PLAT432_ALERT_2_G Short Inter X...Y Contact  C2     ..  C14X    ..       3.04 Ang.
PLAT432_ALERT_2_G Short Inter X...Y Contact  C3     ..  C13X    ..       2.69 Ang.
PLAT432_ALERT_2_G Short Inter X...Y Contact  C5     ..  C14X    ..       3.00 Ang.
PLAT432_ALERT_2_G Short Inter X...Y Contact  C6     ..  C14X    ..       3.13 Ang.
PLAT432_ALERT_2_G Short Inter X...Y Contact  C7     ..  C14X    ..       2.10 Ang.
PLAT432_ALERT_2_G Short Inter X...Y Contact  C7     ..  C12     ..       2.74 Ang.
PLAT432_ALERT_2_G Short Inter X...Y Contact  C7     ..  C14     ..       2.89 Ang.
PLAT432_ALERT_2_G Short Inter X...Y Contact  C7     ..  C13     ..       3.03 Ang.
PLAT710_ALERT_4_G Delete 1-2-3 or 2-3-4 Linear Torsion Angle ... #          1 Do !
            CL1 -AU1 -AS1 -C8X   -56.80  1.10   1.555   1.555   1.555   8.565
PLAT710_ALERT_4_G Delete 1-2-3 or 2-3-4 Linear Torsion Angle ... #          2 Do !
            CL1 -AU1 -AS1 -C8X    56.80  1.10   1.555   1.555   1.555   1.555
PLAT710_ALERT_4_G Delete 1-2-3 or 2-3-4 Linear Torsion Angle ... #          3 Do !
            CL1 -AU1 -AS1 -C1    180.00  0.00   1.555   1.555   1.555   1.555
PLAT710_ALERT_4_G Delete 1-2-3 or 2-3-4 Linear Torsion Angle ... #          4 Do !
            CL1 -AU1 -AS1 -C8    -62.90  1.10   1.555   1.555   1.555   8.565
PLAT710_ALERT_4_G Delete 1-2-3 or 2-3-4 Linear Torsion Angle ... #          5 Do !
            CL1 -AU1 -AS1 -C8     62.90  1.10   1.555   1.555   1.555   1.555
PLAT720_ALERT_4_G Number of Unusual/Non-Standard Labels ..........          1 Note
PLAT764_ALERT_4_G Overcomplete CIF Bond List Detected (Rep/Expd) .       1.35 Ratio
PLAT773_ALERT_2_G Check long C-C Bond in CIF: C2     --  C5      .       1.96 Ang.
PLAT773_ALERT_2_G Check long C-C Bond in CIF: C3     --  C6      .       1.77 Ang.
PLAT773_ALERT_2_G Check long C-C Bond in CIF: C5     --  C5      .       1.85 Ang.
PLAT773_ALERT_2_G Check long C-C Bond in CIF: C5     --  C2      .       1.96 Ang.
PLAT773_ALERT_2_G Check long C-C Bond in CIF: C6     --  C3      .       1.77 Ang.
PLAT773_ALERT_2_G Check long C-C Bond in CIF: C6     --  C6      .       1.96 Ang.
PLAT779_ALERT_4_G Suspect or Irrelevant (Bond) Angle in CIF .... #          5 Check
              C8X  -AS1  -C8      8.565   1.555   8.565             7.40 Deg.
PLAT779_ALERT_4_G Suspect or Irrelevant (Bond) Angle in CIF .... #          9 Check
              C8X  -AS1  -C8      1.555   1.555   1.555             7.40 Deg.
PLAT779_ALERT_4_G Suspect or Irrelevant (Bond) Angle in CIF .... #         32 Check
              C3   -C2   -C5      1.555   1.555   8.565            38.50 Deg.
PLAT779_ALERT_4_G Suspect or Irrelevant (Bond) Angle in CIF .... #         41 Check
              C2   -C3   -C6      1.555   1.555   8.565            44.20 Deg.
PLAT779_ALERT_4_G Suspect or Irrelevant (Bond) Angle in CIF .... #         64 Check
              C4   -C5   -C5      1.555   1.555   8.565            43.10 Deg.
PLAT779_ALERT_4_G Suspect or Irrelevant (Bond) Angle in CIF .... #         69 Check
              C6   -C5   -C2      1.555   1.555   8.565            39.10 Deg.
PLAT779_ALERT_4_G Suspect or Irrelevant (Bond) Angle in CIF .... #         77 Check
              C5   -C6   -C3      1.555   1.555   8.565            43.70 Deg.
PLAT779_ALERT_4_G Suspect or Irrelevant (Bond) Angle in CIF .... #         79 Check
              C1   -C6   -C6      1.555   1.555   8.565            44.00 Deg.
PLAT811_ALERT_5_G No ADDSYM Analysis: Too Many Excluded Atoms ....          ! Info
PLAT910_ALERT_3_G Missing # of FCF Reflections Below Th(Min) .....          1 Report
```

```
 0 ALERT level A = Most likely a serious problem - resolve or explain
 0 ALERT level B = A potentially serious problem, consider carefully
 6 ALERT level C = Check. Ensure it is not caused by an omission or oversight
38 ALERT level G = General information/check it is not something unexpected

 2 ALERT type 1 CIF construction/syntax error, inconsistent or missing data
20 ALERT type 2 Indicator that the structure model may be wrong or deficient
 4 ALERT type 3 Indicator that the structure quality may be low
17 ALERT type 4 Improvement, methodology, query or suggestion
 1 ALERT type 5 Informative message, check
```

checkCIF publication errors

Alert level A

```
PUBL024_ALERT_1_A The number of authors is greater than 5.
              Please specify the role of each of the co-authors
              for your paper.
```

Author Response: The design of experiment and synthesis and spectral characterization work was conducted by the visiting scholar Tariq with Khan assistance. The crystal structures solving and refinement were carried out by both Goh and Rosli. Final verification and justification of work were confirmed by both Shawkataly and Fun.

Alert level G

```
PUBL017_ALERT_1_G The _publ_section_references section is missing or
            empty.
```

```
 1 ALERT level A = Data missing that is essential or data in wrong format
 1 ALERT level G = General alerts. Data that may be required is missing
```

Publication of your CIF

You should attempt to resolve as many as possible of the alerts in all categories. Often the minor alerts point to easily fixed oversights, errors and omissions in your CIF or refinement strategy, so attention to these fine details can be worthwhile. In order to resolve some of the more serious problems it may be necessary to carry out additional measurements or structure refinements. However, the nature of your study may justify the reported deviations from journal submission requirements and the more serious of these should be commented upon in the discussion or experimental section of a paper or in the "special_details" fields of the CIF. *checkCIF* was carefully designed to identify outliers and unusual parameters, but every test has its limitations and alerts that are not important in a particular case may appear. Conversely, the absence of alerts does not guarantee there are no aspects of the results needing attention. It is up to the individual to critically assess their own results and, if necessary, seek expert advice.

If you wish to submit your CIF for publication in Acta Crystallographica Section C or E, you should upload your CIF via the web. If your CIF is to form part of a submission to another IUCr journal, you will be asked, either during electronic submission or by the Co-editor handling your paper, to upload your CIF via our web site.

PLATON version of 24/07/2014; check.def file version of 24/07/2014

Datablock I - ellipsoid plot

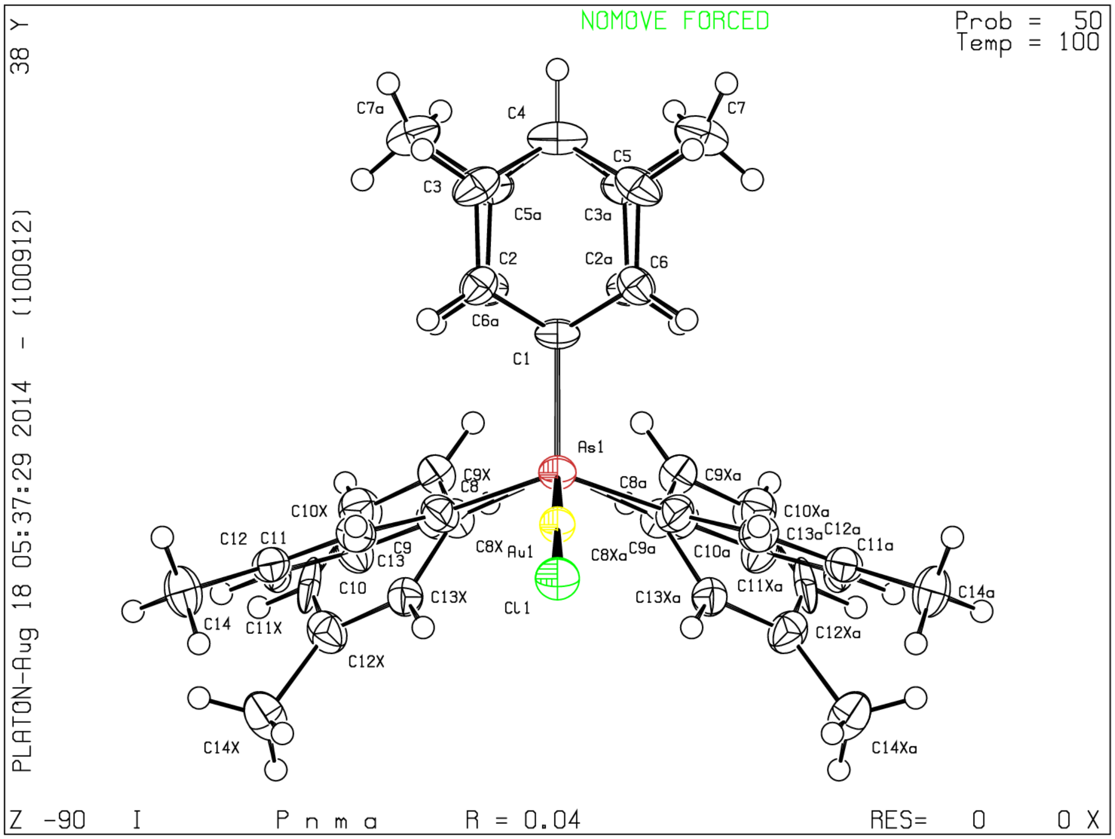

Supplement: S2 Text — (PDF) [file pone.0119620.s006.pdf]
