# Supplementary material for: Synthesis, Spectral Characterization and Crystals Structure of some Arsane Derivatives of Gold (I) Complexes: A Comparative Density Functional Theory Study
Source: PLoS One. 2015 Mar 23;10(3):e0119620. doi: 10.1371/journal.pone.0119620 (PMC4370652; doi:10.1371/journal.pone.0119620)

checkCIF/PLATON report

Structure factors have been supplied for datablock(s) I

THIS REPORT IS FOR GUIDANCE ONLY. IF USED AS PART OF A REVIEW PROCEDURE FOR PUBLICATION, IT SHOULD NOT REPLACE THE EXPERTISE OF AN EXPERIENCED CRYSTALLOGRAPHIC REFEREE.

No syntax errors found. CIF dictionary Interpreting this report

Datablock: I

Bond precision: C-C = 0.0030 A Wavelength=0.71073

Cell:	a=9.4715(3)	b=18.0504(5)	c=16.0002(5)
	alpha=90	beta=96.461(1)	gamma=90
Temperature:	100 K		

	Calculated	Reported
Volume	2718.09(14)	2718.09(14)
Space group	P 21/c	P2(1)/c
Hall group	-P 2ybc	?
Moiety formula	C30 H21 As Au Cl, C H2 Cl2	?
Sum formula	C31 H23 As Au Cl3	C20.67 H15.33 As0.67 Au0.67 Cl2
Mr	773.73	515.82
Dx,g cm-3	1.891	1.891
Z	4	6
Mu (mm-1)	6.935	6.935
F000	1488.0	1488.0
F000’	1482.69	
h,k,lmax	16,30,27	16,30,27
Nref	14227	14091
Tmin,Tmax	0.253,0.500	0.133,0.559
Tmin’	0.032	

Correction method= MULTI-SCAN

Data completeness= 0.990 Theta(max)= 37.400

R(reflections)= 0.0287(11628) wR2(reflections)= 0.0665(14091)

S = 1.018 Npar= 325

The following ALERTS were generated. Each ALERT has the format
test-name_ALERT_alert-type_alert-level.
Click on the hyperlinks for more details of the test.

Alert level B

```
PLAT971_ALERT_2_B Check Calcd Residual Density  0.81A From     Au1       3.17 eA-3
PLAT971_ALERT_2_B Check Calcd Residual Density  0.88A From     Au1       3.03 eA-3
```

Alert level C

```
ABSTY02_ALERT_1_C  An _exptl_absorpt_correction_type has been given without
            a literature citation. This should be contained in the
            _exptl_absorpt_process_details field.
            Absorption correction given as multi-scan
PLAT048_ALERT_1_C MoietyFormula Not Given ........................     Please Do !
PLAT094_ALERT_2_C Ratio of Maximum / Minimum Residual Density ....       2.69 Report
PLAT125_ALERT_4_C No '_symmetry_space_group_name_Hall' Given .....     Please Do !
PLAT244_ALERT_4_C Low   'Solvent' Ueq as Compared to Neighbors of        C31 Check
PLAT911_ALERT_3_C Missing # FCF Refl Between THmin & STh/L=  0.600        63 Report
PLAT913_ALERT_3_C Missing # of Very Strong Reflections in FCF ....         4 Note
```

Alert level G

```
CELLZ01_ALERT_1_G Difference between formula and atom_site contents detected.
CELLZ01_ALERT_1_G ALERT: check formula stoichiometry or atom site occupancies.
           From the CIF: _cell_formula_units_Z    6
           From the CIF: _chemical_formula_sum  C20.67 H15.33 As0.67 Au0.67 Cl2
           TEST: Compare cell contents of formula and atom_site data

           atom    Z*formula  cif sites diff
           C        124.02    124.00    0.02
           H         91.98     92.00   -0.02
           As         4.02      4.00    0.02
           Au         4.02      4.00    0.02
           Cl        12.00     12.00    0.00
PLAT045_ALERT_1_G Calculated and Reported Z Differ by ...........       0.67 Ratio
PLAT093_ALERT_1_G No su's on H-positions, refinement reported as .      mixed
PLAT710_ALERT_4_G Delete 1-2-3 or 2-3-4 Linear Torsion Angle ... #         1 Do !
            CL1 -AU1 -AS1 -C10   -68.40  0.40   1.555   1.555   1.555   1.555
PLAT710_ALERT_4_G Delete 1-2-3 or 2-3-4 Linear Torsion Angle ... #         2 Do !
            CL1 -AU1 -AS1 -C30   169.70  0.40   1.555   1.555   1.555   1.555
PLAT710_ALERT_4_G Delete 1-2-3 or 2-3-4 Linear Torsion Angle ... #         3 Do !
            CL1 -AU1 -AS1 -C20    49.10  0.40   1.555   1.555   1.555   1.555
PLAT912_ALERT_4_G Missing # of FCF Reflections Above STh/L=  0.600        77 Note
```

---

0 **ALERT level A** = Most likely a serious problem - resolve or explain
2 **ALERT level B** = A potentially serious problem, consider carefully
7 **ALERT level C** = Check. Ensure it is not caused by an omission or oversight
8 **ALERT level G** = General information/check it is not something unexpected

0 ALERT type 1 CIF construction/syntax error, inconsistent or missing data
3 ALERT type 2 Indicator that the structure model may be wrong or deficient
2 ALERT type 3 Indicator that the structure quality may be low
6 ALERT type 4 Improvement, methodology, query or suggestion
0 ALERT type 5 Informative message, check

---

checkCIF publication errors

Alert level A

```
PUBL024_ALERT_1_A The number of authors is greater than 5.
              Please specify the role of each of the co-authors
              for your paper.
```

Author Response: The design of experiment and synthesis and spectral characterization work was conducted by the visiting scholar Tariq with Khan assistance. The crystal structures solving and refinement were carried out by both Goh and Rosli. Final verification and justification of work were confirmed by both Shawkataly and Fun.

Alert level G

```
PUBL017_ALERT_1_G The _publ_section_references section is missing or
            empty.
```

---

1 **ALERT level A** = Data missing that is essential or data in wrong format
1 **ALERT level G** = General alerts. Data that may be required is missing

---

Publication of your CIF

You should attempt to resolve as many as possible of the alerts in all categories. Often the minor alerts point to easily fixed oversights, errors and omissions in your CIF or refinement strategy, so attention to these fine details can be worthwhile. In order to resolve some of the more serious problems it may be necessary to carry out additional measurements or structure refinements. However, the nature of your study may justify the reported deviations from journal submission requirements and the more serious of these should be commented upon in the discussion or experimental section of a paper or in the "special_details" fields of the CIF. *checkCIF* was carefully designed to identify outliers and unusual parameters, but every test has its limitations and alerts that are not important in a particular case may appear. Conversely, the absence of alerts does not guarantee there are no aspects of the results needing attention. It is up to the individual to critically assess their own results and, if necessary, seek expert advice.

If you wish to submit your CIF for publication in Acta Crystallographica Section C or E, you should upload your CIF via the web. If your CIF is to form part of a submission to another IUCr journal, you will be asked, either during electronic submission or by the Co-editor handling your paper, to upload your CIF via our web site.

PLATON version of 24/07/2014; check.def file version of 24/07/2014

Datablock I - ellipsoid plot

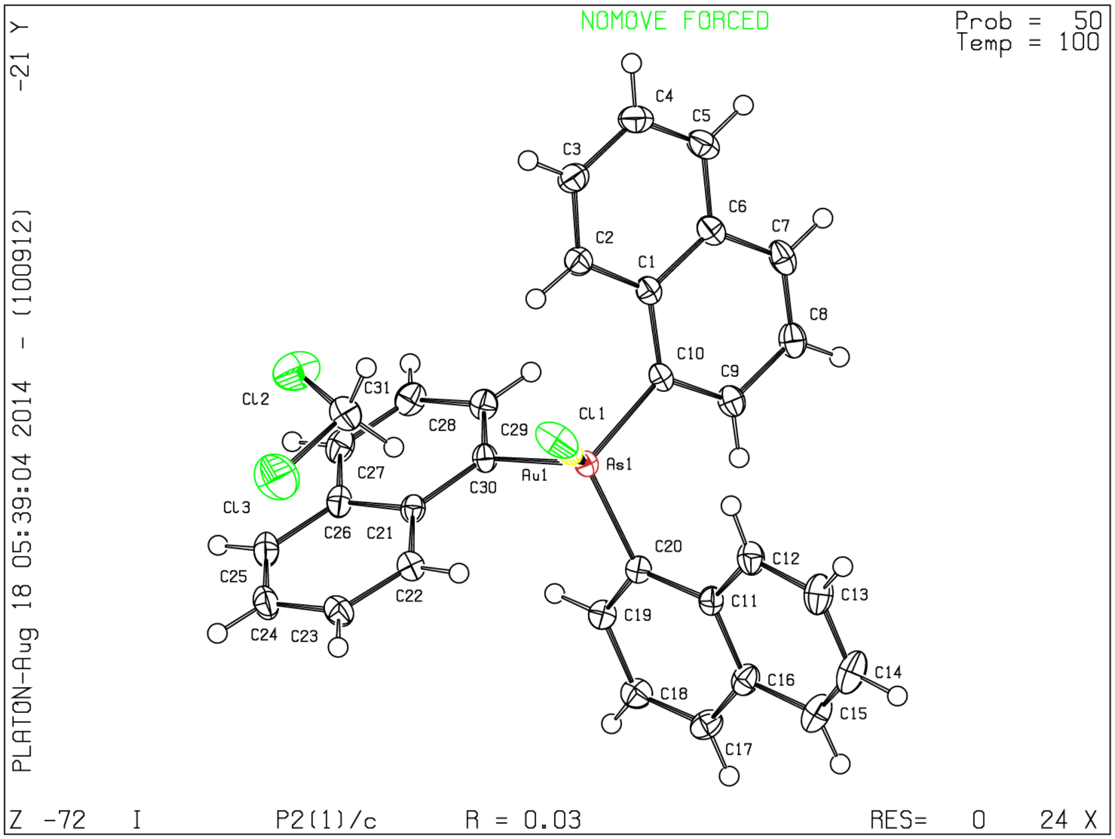

Supplement: S4 Text — (PDF) [file pone.0119620.s008.pdf]
