# Supplementary material for: Synthesis, Spectral Characterization and Crystals Structure of some Arsane Derivatives of Gold (I) Complexes: A Comparative Density Functional Theory Study
Source: PLoS One. 2015 Mar 23;10(3):e0119620. doi: 10.1371/journal.pone.0119620 (PMC4370652; doi:10.1371/journal.pone.0119620)

checkCIF/PLATON report

Structure factors have been supplied for datablock(s) I

THIS REPORT IS FOR GUIDANCE ONLY. IF USED AS PART OF A REVIEW PROCEDURE FOR PUBLICATION, IT SHOULD NOT REPLACE THE EXPERTISE OF AN EXPERIENCED CRYSTALLOGRAPHIC REFEREE.

No syntax errors found. CIF dictionary Interpreting this report

Datablock: I

```
Bond precision:  C-C = 0.0168 A          Wavelength=0.71073

Cell:            a=12.9884(9)     b=17.0042(12)      c=13.3251(9)
                 alpha=90         beta=90.318(2)     gamma=90
Temperature:     100 K
```

	Calculated	Reported
Volume	2942.9(4)	2942.9(4)
Space group	P 21	P 21
Hall group	P 2yb	P 2yb
Moiety formula	C21 H21 As Au Cl	C21 H21 As Au Cl
Sum formula	C21 H21 As Au Cl	C21 H21 As Au Cl
Mr	580.72	580.71
Dx,g cm-3	1.966	1.966
Z	6	6
Mu (mm-1)	9.308	9.308
F000	1656.0	1656.0
F000’	1646.38	
h,k,lmax	15,20,15	15,20,15
Nref	10362[5373]	10193
Tmin,Tmax	0.088,0.118	0.108,0.227
Tmin’	0.016	

```
Correction method= MULTI-SCAN

Data completeness= 1.90/0.98      Theta(max)= 25.000

R(reflections)= 0.0427( 9939)     wR2(reflections)= 0.1086( 10193)

S = 1.012                  Npar= 634
```

The following ALERTS were generated. Each ALERT has the format
test-name_ALERT_alert-type_alert-level.
Click on the hyperlinks for more details of the test.

Alert level A

```
PLAT971_ALERT_2_A Check Calcd Residual Density  0.70A From     C8B       6.07 eA-3
```

Author Response: The alert was mainly due to there is q peak that not assigned to any element. However, when observed closely the electron density appears to be around phenyl ring and also near to the metal centres. Thus,such residual density is likely not from unaccounted atom types.

Alert level B

```
PLAT094_ALERT_2_B Ratio of Maximum / Minimum Residual Density ....       5.70 Report
PLAT971_ALERT_2_B Check Calcd Residual Density  0.79A From    Au1C       3.06 eA-3
```

Author Response: The alert was mainly due to there is q peak that not assigned to any element. However, when observed closely the electron density appears to be around phenyl ring and also near to the metal centres. Thus,such residual density is likely not from unaccounted atom types.

Alert level C

```
DIFMX01_ALERT_2_C  The maximum difference density is > 0.1*ZMAX*0.75
            _refine_diff_density_max given =      6.038
            Test value =      5.925
DIFMX02_ALERT_1_C  The maximum difference density is > 0.1*ZMAX*0.75
            The relevant atom site should be identified.
PLAT090_ALERT_3_C Poor Data / Parameter Ratio (Zmax > 18) ........       8.45 Note
PLAT097_ALERT_2_C Large Reported Max.  (Positive) Residual Density       6.04 eA-3
PLAT342_ALERT_3_C Low Bond Precision on  C-C Bonds ...............     0.0168 Ang.
PLAT911_ALERT_3_C Missing # FCF Refl Between THmin & STh/L=  0.595         14 Report
PLAT913_ALERT_3_C Missing # of Very Strong Reflections in FCF ....          4 Note
PLAT971_ALERT_2_C Check Calcd Residual Density  0.90A From    As1B       2.41 eA-3
```

Author Response: The alert was mainly due to there is q peak that not assigned to any element. However, when observed closely the electron density appears to be around phenyl ring and also near to the metal centres. Thus,such residual density is likely not from unaccounted atom types.

```
PLAT971_ALERT_2_C Check Calcd Residual Density  0.85A From    Au1A       1.92 eA-3
```

Author Response: The alert was mainly due to there is q peak that not assigned to any element. However, when observed closely the electron density appears to be around phenyl ring and also near to the metal centres. Thus,such residual density is likely not from unaccounted atom types.

```
PLAT971_ALERT_2_C Check Calcd Residual Density  0.91A From    Au1C       1.58 eA-3
```

Author Response: The alert was mainly due to there is q peak that not assigned to any element. However, when observed closely the electron density appears to be around phenyl ring and also near to the metal centres. Thus,such residual density is likely not from unaccounted atom types.

```
PLAT971_ALERT_2_C Check Calcd Residual Density  0.84A From    Au1A       1.54 eA-3
```

Author Response: The alert was mainly due to there is q peak that not assigned to any element. However, when observed closely the electron density appears to be around phenyl ring and also near to the metal centres. Thus,such residual density is likely not from unaccounted atom types.

Alert level G

```
PLAT003_ALERT_2_G Number of Uiso or Uij Restrained non-H Atoms ...          1 Report
PLAT033_ALERT_4_G Flack x Value Deviates > 2*sigma from Zero .....      0.019
PLAT083_ALERT_2_G SHELXL Second Parameter in WGHT Unusually Large.      27.78 Report
PLAT093_ALERT_1_G No su’s on H-positions, refinement reported as .      mixed
PLAT171_ALERT_4_G The CIF-Embedded .res File Contains EADP Records          2 Report
PLAT720_ALERT_4_G Number of Unusual/Non-Standard Labels ..........         18 Note
PLAT860_ALERT_3_G Number of Least-Squares Restraints .............          7 Note
PLAT909_ALERT_3_G Percentage of Observed Data at Theta(Max) still          92 %
PLAT910_ALERT_3_G Missing # of FCF Reflections Below Th(Min) .....          4 Report
```

```
   1 ALERT level A = Most likely a serious problem - resolve or explain
   2 ALERT level B = A potentially serious problem, consider carefully
  11 ALERT level C = Check. Ensure it is not caused by an omission or oversight
   9 ALERT level G = General information/check it is not something unexpected

   2 ALERT type 1 CIF construction/syntax error, inconsistent or missing data
  11 ALERT type 2 Indicator that the structure model may be wrong or deficient
   7 ALERT type 3 Indicator that the structure quality may be low
   3 ALERT type 4 Improvement, methodology, query or suggestion
   0 ALERT type 5 Informative message, check
```

---

checkCIF publication errors

Alert level A

```
PUBL024_ALERT_1_A The number of authors is greater than 5.
              Please specify the role of each of the co-authors
              for your paper.
```

Author Response: The design of experiment and synthesis and spectral characterization work was conducted by the visiting scholar Tariq with Khan assistance. The crystal structures solving and refinement were carried out by both Goh and Rosli. Final verification and justification of work were confirmed by both Shawkataly and Fun.

Alert level G

```
PUBL017_ALERT_1_G The _publ_section_references section is missing or
            empty.
```

```
   1 ALERT level A = Data missing that is essential or data in wrong format
   1 ALERT level G = General alerts. Data that may be required is missing
```

Publication of your CIF

You should attempt to resolve as many as possible of the alerts in all categories. Often the minor alerts point to easily fixed oversights, errors and omissions in your CIF or refinement strategy, so attention to these fine details can be worthwhile. In order to resolve some of the more serious problems it may be necessary to carry out additional measurements or structure refinements. However, the nature of your study may justify the reported deviations from journal submission requirements and the more serious of these should be commented upon in the discussion or experimental section of a paper or in the "special_details" fields of the CIF. *checkCIF* was carefully designed to identify outliers and unusual parameters, but every test has its limitations and alerts that are not important in a particular case may appear. Conversely, the absence of alerts does not guarantee there are no aspects of the results needing attention. It is up to the individual to critically assess their own results and, if necessary, seek expert advice.

If you wish to submit your CIF for publication in Acta Crystallographica Section C or E, you should upload your CIF via the web. If your CIF is to form part of a submission to another IUCr journal, you will be asked, either during electronic submission or by the Co-editor handling your paper, to upload your CIF via our web site.

PLATON version of 24/07/2014; check.def file version of 24/07/2014

Datablock I - ellipsoid plot

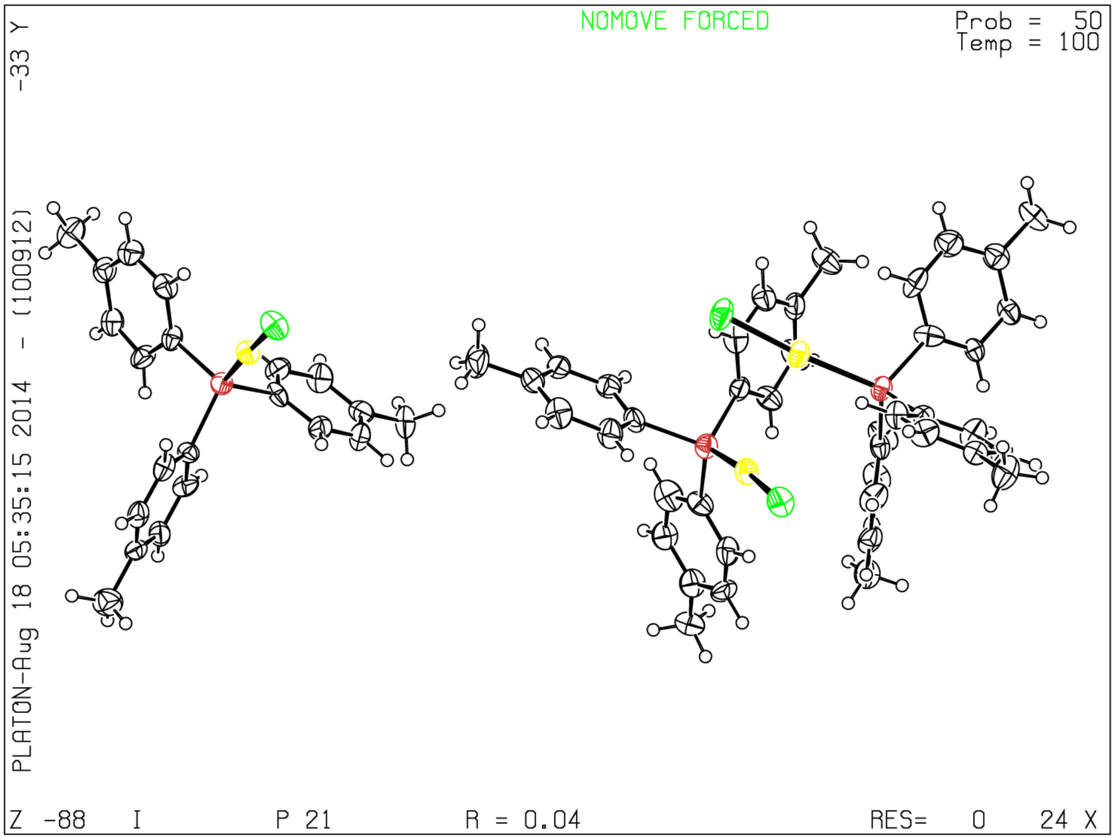

Supplement: S1 Text — (PDF) [file pone.0119620.s005.pdf]
